# The Lower Denture

**Published:** 1903-12-15

**Authors:** R. C. Brophy


### THE LOWER DENTURE.

BY R. C. BROPHY, D.D.S.

Read before the Missouri Dental Society.

The matter of making lower dentures is one which merits special consideration. There is a wide difference between conditions met with and the princlples involved in constructing an upper and a lower denture. In the former, the striking of a nice equilibrium of pressure on the field of contact upon a basis of the hardness and softness met with and the adaptation which remains unbroken by reason of the force of mastication creating undue pressure against the jaw at certain points, and the prevention of undue lifting stress of the muscles of the cheeks and lips upon the rim of the denture, serves to induce its being held rigidly in position through capillary attraction.

In lower dentures this law of attraction cannot be relied upon, though probably all of us have noted its existence in occasional cases, in greater or lesser degree, depending also in these cases upon the perfection of the adaptation or fit of the base of the denture, and the character of the tissues underlying it. In lower dentures dependence for stability of position in the mouth must be expected from other sources—suction, as it is more commonly termed, offering no dependable aid.

It must be conceded that in constructing the lower denture absolute perfection of adaptation of the base to the alveolar ridge is quite as important as would be the case were capillary attractiou depended upon to hold if in place. With perfect adaptation of the base to the process accomplished, next comes the exercise of strategy to evoke mechanical aid in sustaining the plate in position. Much may be done toward aiding the, at best, unfortunate individual who wears an artificial lower denture in gaining the retentive ability to keep it in place. One of the most potent of these aids is to carry the posterio-lingual wings of the denture well downward, and by finishing with marked concavities the lingual surfaces of these wings, tend to aid retention of the plate through the tongue's lying in these grooves or concavities. Great care should be observed in relieving the denture of all impingement tending to lift it from a solid seat on the ridge, the point mesio-lingually particularly demanding attention.

Buccally and labially, it usually is well to finish with the rims somewhat flaring, thus permitting the muscles to aid in holding the denture down by lying over them. It is hardly necessary for me to mention the importance to stability of the lower denture of the manner of occluding the lower with the upper teeth. A denture may fit the process perfectly, but the occlusion of the teeth upon it with the antagonizing teeth above may utterly preclude stability of the denture. Perfect occlusion and correct articulation of the entire posterior area with absence of absolute contact anteriorly is important and should not be overlooked.

The matter of aiding stability of the lower denture by constructing it of material which, through possessing greater weight, brings into service the law of gravitation, is one which it is my disposition to lay before you with particular stress. While it is argued by some men prominent in this department of practice—notably Dr. L. P. Haskell, of Chicago—that weight is of no advantage in the stability of a lower denture, I am forced to declare an opposing mind. In those cases wherein, through excessive absorption, the alveolar ridge has quite disappeared, I am convinced that weight is an advantage, and my own experience has convinced me that in no case is it a disadvantage. I wish to show you some dentures which, as a result of a number of years' experience, I am satisfied offer many decided advantages over every other kind of denture, except continuous gum, and in the matter of the quality of fitting these plates, if made by a method which it will be my pleasure to demonstrate to you have no equal. I would call your attention further to the fact that upper base-plates, made after this same plan, result in perfection of adaptation to the jaw. There is none more compatible to the soft tissues, and it presents no greater weight than does the continuous gum denture.

The plates which I present to you were made of an alloy prepared by myself and which differs from the alloy known as Watt's metal only in that the quantity of tin is reduced and other metals substituted, which results in a more rigid and whiter alloy which is much less subject to tarnishing and discoloration in the mouth.

The manner of constructing these base-plates differs but slightly from the method generally followed of manipulating

Watts' or Weston's metals, this slight difference, however, comprehends the perfection of fitting which I claim for my method of casting this class of base-plates, and it is, therefore, to this difference that your attention is specially directed.

It is a well-known fact that plaster of paris is incapable of withstanding the application of extreme heat without marked change in bulk. It ought readily to be understood, therefore, that to undertake to cast base-plates or dentures to a plaster model, which necessarily must be subjected to heat in the process, invites failure of the plates fitting when placed in the mouth. To be brief, then, I would say to cast a base-plate that will fit the mouth, it must be cast to a model which is not perceptibly changed from perfect duplication of the form of the jaw upon which the plate is to rest. Let me explain my method of accomplishing this result.

An impression is taken, and it is absolutely imperative that the impression be a perfect one, in order that a perfect-fitting plate may be secured. After the impression is taken a very thin shellac varnish is applied to it and after it has dried, the surface should be soaped—that is, a fine brush should be wet and then passed over a bar of soap until a slight lather is produced, when the surface of the impression should be carefully gone over with the brush. Plaster of paris should now be mixed thinly and poured into the impression, and immediately out again; this to be repeated until a film or coating of plaster not to exceed a thirty-second of an inch in thickness shall completely and evenly cover the surface of the impression. Imperial investment compound is now mixed to a stiff, putty-like consistency and immediately placed in the plaster-coated impression. It is important that this be done before the plaster forming the coating sets to dryness, for in that case the coating would be liable to flake off the model either in the process of separating or subsequently. This process of coating the impression with plaster is for the purpose of forming a veneering over the model to give it a smooth surface, the investment material being of too coarse a character to give the plate, if cast direct to it, a surface sufficiently smooth.

This gives to us a model which is unchangeable under heat, except for the infinitesimal shrinkage in the thin plaster veneering, which, if worthy of consideration at all, would not be likely to be found a disadvantage. After the model is prepared, then the ordinary procedure, generally known, of casting Watts' metal plates, is followed, except that the case should be invested in the flank with the same investment compound of which the model is made. When this is done, the fact that the compound is non-shrinkable insures the retention of the molten metal within the matrix.

It will be found that full and partial dentures may be made after this mode, casting direct to the teeth, using no vulcanite, or, if desired, only a layer of pink vulcanite anteriorly. I am opposed to the use of vulcanite for base-plates. Several years ago I made my last vulcanite plate, and I shall never make another one. I am satisfied that God did not make vulcanite to be used for any such purpose. I believe it to be contrary to Nature's laws to expect animal tissue to thrive beneath a plate of vegetable matter, devoid of free conductivity of heat where the contact remains unbroken so constantly as is the case of upper plates. Vulcanite base-plates worn any considerable time always induce an abnormal condition of the tissues underlying them.

You will note that I have referred to the use of vulcanite for *upper* plates. I concede that, owing to the difference in the nature of the contact, this material is much less objectionable for use in lower dentures; but I am disposed to proscribe it, on general principles, for all cases. You will furthermore note that I have offered no objection to vulcanite on the ground that it is a poisonous material or substance. I do not do this, because I do not think that this is responsible for the objectionable results of its use. The use of metal in constructing base-plates for dentures, and the abandonment of vulcanite for that purpose, is demanded in the interests of humanity and for the credit of our profession as a scientific body.

DISCUSSION.

Dr. J. B. Wilmott: So far as the non-conducting properties are concerned, it has never struck me that we gained anything by making a vulcanite attachment for a lower denture. In an upper denture we do, over that portion of the plate which is not covered by vulcanite, but just to the extent the lower rim of that kind is covered by vulcanite, we destroy the conducting properties of the metal as far as carrying away heat from the tissues is concerned, precisely to the same extent as though made entirely of vulcanite. Whether vulcanite itself is an irritant, is another question; my own impression is that it is not; that the tissues will bear a clean vulcanite plate when properly vulcanized, and, especially if it is not red, tolerate it just as well as they will metal. The only objection at all from my standpoint would be, it is undoubtedly a non-conductor and the tissues in many mouths become overheated because the natural heat is not reduced by the cooling effect of radiation in the air.

Dr. J. D. Patterson: My experience is that in many mouths it has been impossible to wear the lower denture with any satisfaction whatever. However perfect the fit, however well the models have been treated, however well the reciprocal pressure on hard and soft parts has been provided for, however perfectly the teeth are placed in the arch, and however well antagonized according to the Bonwill method against the opposing teeth, yet the denture could not be worn satisfactorily. With the added weight of a metal base, the plate is worn with almost entire satisfaction, which it seems could not be procured in any way save by the weight. I have heard of patients to whom that added weight was a disadvantage. I have never experienced that objection myself after a plate has been worn for a few weeks. If a heavy plate has been substituted for a lighter one, the added weight for a few days or weeks is noted by the patient, and causes discomfort, but after a time they ceased to notice it. I think the use of added weight solves the difficulty which we often find in a lower denture, such as where there is an almost complete absence of the ridge, the additional gravitation of this added weight is the only solution that I have found.

No one has spoken about the finishing of the plate after it is prepared. This metal I presume, as well as the Watts' metal, can be gold-plated most beautifully, and the plate will look more cleanly. The plating does not interfere with the fit and it makes a beautiful plate which will not tarnish, as the plain base metal will do in time.

I had an opportunity at one time to test the point of how much the lower mandible would stand in the way of weight, in an edentulous mouth, before the wearing of the plate would become tiresome, and I found that this person could conveniently wear a plate, without that weight becoming burdensome, up to 50 pennyweights, but that anything over that weight was a burden. Of course, that experience would not work out in all mouths, because such a plate might prove to be very disagreeable in another mouth. The usual plate made with Watts' metal, if no rubber attachment is used, will weigh from 25 to 28 pennyweights, and that is quite heavy enough. To those who have complaints regarding the weight of plates, I would say if they will insist upon the patients wearing the plates for two or three months, the objections will have passed away.

Dr. J. F. Wallace: We should take pains to extend the lingual margins of the plate equidistant posteriorly. The muscles of the tongue interfere very little in that part of the mouth with an extended plate. You will find in examining the lower maxilla that at that part the space across the mouth is narrower at the top than it is at the bottom of the bone. Only a few days ago I fitted a plate where the upper portion of the ridge was almost entirely absorbed, there was not enough to keep the plate from jostling around; I extended it quite a little in the posterior portion and when I put the plate in, instead of dropping it down on the ridge, I had to pass it back, and draw it forward a little before it dropped down. My patient was back one day to have the plate trimmed a little anteriorly, but the posterior portion gave her no annoyance and it added very materially to the stability.

Dr. W. M. Bartlett: Dr. Brophy suggests the use of shellac varnish and over the surface of this varnish to use a coating of soap. I believe that when Dr. Brophy places upon the surface of an impression a film of gum shellac, that he there reduces to some extent the caliber of that impression, and by placing upon this surface another layer of soap, he again reduces it, giving him to some extent a faulty margin. I would suggest the use of shellac dissolved in a saturated solution of borax water, which makes a most excellent separating medium; it is obtained by making a saturated solution of borax, and dissolving the shellac in this, allowing the saturated solution of borax water to take up as much shellac as possible; this being an aqueous solution, it is readily absorbed by the plaster of paris, and produces no covering of shellac on the surface of the impression. This can be placed upon the plaster, and separates with perfect ease, giving a smooth surface to the impression.

I believe that if we have a good impression of the lower jaw, we can construct a denture of vulcanite or any other material, taking the occlusion, the curve and the position of the arch into account, that will give entire satisfaction to the patient.

As far as sore mouths are concerned, we find sore mouths under every base excepting platinum when thoroughly constructed and adapted to the ridges and to the palatal vault. If it has a faulty adaptatiou and an imperfect finish of the palatal surface you will have sore mouths. This "hoboe" business of the mercury in the rubber is played out. We find it in cheoplastic work of all kinds, under gold, under silver (if any of us are mean enough to make a silver base), but you do not find it under a perfect platinum plate.

Dr. B. Q. Stevens: In getting a model from the plaster cast—I always take my impressions in plaster, and I never coat them with anything at all—if you want a perfect impression, take your plaster cast and set it in water about ten minutes and pour your plaster in the impression.

You have no trouble that way; they will separate perfectly without the use of varnish or anything else, and the model will not be marred.

Dr. C. L. Hungerford: I should be glad of some method of separating that was satisfactory. I have heard one illustration of a means to that end this afternoon that I hope will be successful, but if it is, it is contrary to every known law of chemistry that I have ever heard of, that is, that gum shellac is soluble in water, borax water, or any other material of which water is the solvent.

One gentleman says that a heavy plate is just as easily displaced by the tongue as a light one. He might as well say that he could blow a cannon-ball from this table as easily as he could a feather. I grant you that we can put no weight in the mouth that the tongue perhaps could not move, but every additional pennyweight of weight will add that much to the stability of the lower denture.

We have heard that gum tissue does not become inflamed under platinum; but it does under other metals that are covered with their oxides or their sulphides. We hear of a silver plate oxidizing. Silver plates do not oxidize; nobody ever saw an oxidized silver plate. You do not see oxidized gold plates much; sometimes a trifle of oxidation on a gold plate. It would not be the salts, though, of the various metals that would produce irritation under metallic plates, and it is their ill-adaptation, rather than the character of the metal, that causes the trouble. The gum tissue will tolerate platinum just as much as it will gold or any other metal that is readily acted upon by the secretions of the mouth, and no better.

Dr. Bartlett: If a resinous gum could not be dissolved in an aqueous solution, I would not be fool enough to get before this body and tell them that it could be.

As far as the oxide of platinum is concerned, I never heard of it, unless we produce that oxide by the flow of oxygen upon the heated platinum base. As to the silver I should have said sulphide.

Dr. Hermann Prinz: All gums and all resins will dissolve in an alkaline medium, and the contrary that shellac cannot be dissolved in water when borax is added is demonstrated to all of you every day in the use of a patent shoe polish.

In regard to the metals and their physiological action upon tissue of the mouth, I can only reiterate the statement of Dr. Bartlett that there is no metal that can be so comfortably borne in the mouth as platinum, and next to it is silver. Why do the greatest surgeons advocate the use of silver sutures, or the use of silver salts? Because these silver salts are the very best means of sterilizing wounds; and no other salt can be so easily produced, and that is so effective, as the silver salt, or, in combination with Watts' metal, silver-tin salts.

Oxide of platinum can never exist in the mouth, for the reason that platinum, being one of the so-called basic metals, is not easily deprived of its oxygen. Oxide of silver does not form in the mouth, but the salt that is formed on a silver plate in the mouth is the hydrate of silver. Oxygen may form on a combination of silver and tin a chloride of silver and chloride of tin which is a salt that keeps the tissues of the mouth in the very best condition under a well-fitting combination metal plate, so that a well-fitting plate constructed of an alloy which contains silver and tin, with other metals added, would constitute an ideal plate, so far as the physiology of the mouth is concerned.

Dr. D. C. Lindsley: I simply want to say, in reply to Dr. Hungerford, that I made this solution six years ago, and have used nothing else besides that solution for the separating of plaster models. It is readily absorbed into the plaster and leaves no film on the cast, as you can readily see when you cut down to the line of demarkation. When you want to separate your impression before the plaster dries on the base portion, you can immediately pour this solution upon it, and in that way save considerable valuable time.—*Western Dental Journal.*

